# Ist die Strahlentherapie beim diffusen großzelligen B-Zell-Lymphom obsolet? Eine radioonkologische Betrachtung der UNFOLDER-Studie

**DOI:** 10.1007/s00066-023-02177-4

**Published:** 2023-11-14

**Authors:** Michael Oertel, Hans Theodor Eich

**Affiliations:** Klinik für Strahlentherapie – Radioonkologie, Universitätsklinikum Münster (UKM), Albert-Schweitzer-Campus 1, Gebäude A1, 48149 Münster, Deutschland

## Hintergrund

Die Rolle der konsolidierenden Radiotherapie (RT) in der Behandlung des diffusen großzelligen B‑Zell-Lymphoms (DLBCL) wird kontrovers diskutiert [1]. Traditionell basiert die Indikationsstellung auf prächemotherapeutischen Risikofaktoren wie einem extralymphatischen Befall oder einem Bulk (in Deutschland i. d. R. definiert als maximaler Befallsdurchmesser ≥ 7,5 cm; [2, 3]). Neuere Studien verfolgen einen PET-stratifizierten Ansatz, bei dem die RT auf PET-positive Restbefunde limitiert wird [4]. Angesichts der heterogenen Datenlage liefert die nun voll publizierte UNFOLDER-Studie zusätzliche Informationen [5].

## Methode und Patientengut

Zwischen Januar 2006 und November 2015 wurden Patienten im Alter von 18 bis 60 Jahren mit einem aggressiven B‑Zell-Lymphom rekrutiert, die entweder genau einen Risikofaktor gemäß altersadjustiertem internationalem Prognoseindex (aaIPI) aufwiesen (erhöhte LDH, ECOG-Status 2 oder 3, Ann-Arbor-Stadium III oder IV) oder bei aaIPI = 0 einen initialen Bulkbefall hatten. Es fand eine Randomisierung in einem 2 × 2-Design statt, bei dem einerseits die Akzelerierung der Chemotherapie (zweiwöchentliche vs. dreiwöchentliche Immunchemotherapie mit Rituximab, Cyclophosphamid, Hydroxydaunorubicin, Vincristin und Prednisolon [R-CHOP]) sowie die Rolle der RT untersucht wurde. In einem Arm wurde eine konsolidierende Involved-field-RT mit 39,6 Gy bei Patienten mit Bulk bzw. extralymphatischem Befall durchgeführt, während in einem anderen auf sie verzichtet wurde.

Insgesamt wurden 700 Patienten mit einem medianen Alter von 47 Jahren eingeschlossen, von denen 89 % ein DLBCL hatten. Bezüglich des Risikoprofils wiesen 83 % einen aaIPI von 1 auf, 42 % eine erhöhte LDH, 57 % einen Bulk und 47 % einen extralymphatischen Befall. Die mediane Nachbeobachtung betrug 66 Monate.

## Ergebnisse

Der Einsatz der RT war mit einer signifikanten Verbesserung des ereignisfreien Überlebens (EFS) nach 3 Jahren verbunden, nämlich 84 % mit RT (95 %-Konfidenzintervall [CI] 80–89 %) vs. 68 % ohne RT (95 %-CI 61–76 %; *p* = 0,0012). Dies ergab sich vor allem durch die im RT-Arm erhöhte Rate von Komplettremissionen (90 % vs. 79 % im Beobachtungsarm) und erniedrigte Rate von partiellen Remissionen (2 % vs. 11 %).

Sowohl bezüglich des progressionsfreien (PFS) als auch des Gesamtüberlebens (OS) waren nach 3 Jahren keine signifikanten Unterschiede festzustellen (PFS: 89 % vs. 81 %; *p* = 0,22; OS: 93 % vs. 93 %; *p* = 0,51). Dies wurde in Subgruppenanalysen der einzelnen Behandlungsarme sowie bei getrennter Betrachtung von extralymphatischem Befall bzw. Patienten in Komplettremission bestätigt. Die RT wurde in der Regel gut vertragen mit 1–3 % Toxizitäten von Grad 3–4.

## Schlussfolgerungen der Autoren

Eine konsolidierende RT führte bei Patienten mit DLBCL im intermediären Risikoprofil zur Verbesserung des EFS ohne Einfluss auf PFS oder OS. Insofern bliebe die Rolle der RT bei diesen Patienten noch zu bestimmen.

## Kommentar

Aus unserer Sicht sind folgende Aspekte für die Diskussion relevant:

- Die alleinige Verbesserung des EFS wirkt auf den ersten Blick ernüchternd. Zu berücksichtigen ist jedoch, dass es sich hierbei um den primären Endpunkt handelte, auf den die Studie gepowert war. Es handelte sich um einen Kompositendpunkt, definiert als Zeit bis zu einem der folgenden Ereignisse: Progression unter Therapie, keine Veränderung, Beendigung der Therapie wegen Toxizität ohne Komplettremission, keine Komplettremission am Ende der Therapie, Rezidiv nach Komplettremission, Tod (jeder Ursache) oder Applikation einer zusätzlichen Therapie. Dies macht die Interpretation herausfordernd.
- Bereits in einer Interimsanalyse hatte sich die Verbesserung des EFS durch den Einsatz der RT gezeigt, was zum vorzeitigen Schluss der Studienarme ohne RT führte; entsprechend sind diese mit jeweils 81 Patienten nur ca. halb so groß wie die RT-Arme (150 bzw. 155 Patienten). Anschließend wurde die zusätzliche RT in den Beobachtungsarmen nicht mehr als zusätzliche Therapie gezählt, sodass im Grunde ein Cross-over der Studienarme vorlag.
- Von den 700 eingeschlossenen Patienten wurden 229 als nicht bestrahlungsfähig eingestuft und separat analysiert. Protokollgemäß wurden extralymphatische Befälle in Knochenmark, Lunge, Leber, Nieren, Dünndarm und Kolon sowie Aszites, Pleura- und Perikardergüsse nicht bestrahlt.
- Eine Subanalyse zum Einfluss der unterschiedlichen extralymphatischen Befälle ist kaum möglich, da sich hier ein breites Spektrum zeigte: Bei den bestrahlbaren Patienten dominierten ossäre (11–14 %) und Weichteilbefälle (11–19 %).
- Allgemein weist das analysierte Patientenkollektiv mit einem aaIPI = 0–1 eine gute Langzeitprognose auf. Bei Patienten mit aaIPI = 0 ohne Bulkbefall konnte die FLYER-Studie eine Nichtunterlegenheit von 4 × R-CHOP + 2 Zyklen alleiniges Rituximab gegenüber dem intensiveren 6 × R-CHOP zeigen, mit einem resultierenden 3‑Jahres-PFS von 96 % [6].
- Rückschlüsse auf ältere Patienten [2] oder junge Hochrisikopatienten mit erhöhtem aaIPI [7] lassen sich aus der UNFOLDER-Studie gemäß den Einschlusskriterien nicht ableiten.
- Eine Durchführung einer PET-CT war nicht Studienbestandteil. Trotzdem wurden 130 FDG-PET Untersuchungen durchgeführt; vor allem in den RT-Armen (70,1 %) und meist zum Restaging nach Systemtherapie (76 %). In insgesamt 13 Fällen habe sich durch die Ergebnisse die Therapieentscheidung geändert.
- Der metabolische Status nach Systemtherapie gewinnt zunehmende Bedeutung: Eine gepoolte Analyse von Patienten mit DLBCL aus British Columbia zeigte, dass bei PET-positivem Restbefund nach R‑CHOP eine signifikante Verschlechterung des (Gesamt‑)Überlebens zu erwarten ist (3-Jahres-OS: 87 % vs. 64 %). Eine nachfolgende RT konnte diese prognostische Verschlechterung nahezu kompensieren [4]. Dem trägt auch die Deutsche S3-Leitlinie Rechnung, nach der Patienten mit PET-positivem Restbefund nachbestrahlt werden sollen. Wichtig: Eine Empfehlung für Patienten mit PET-negativem Befund gibt es nicht, sodass hier eine konsolidierende RT oder die Nachsorge möglich ist [8].
- Allgemein ist gegenüber einem dichotomen Vorgehen (RT „ja“ oder „nein“) eine differenzierte und risikoadaptierte Indikationsstellung vorzuziehen, die vor Kurzem in einem Editorial des *Red Journals* skizziert wurde [9], Kommentar: [10].

### Schlussfolgerung

Aufgrund der genannten Einschränkungen und des vorzeitigen Rekrutierungsstopps der Nichtbestrahlungsarme ist eine vorsichtige Interpretation der Studienergebnisse der UNFOLDER-Studie notwendig. Zusammenfassend sollte die Indikation zur RT individualisiert erfolgen und hierbei Patientenalter, aaIPI, prächemotherapeutische Risikofaktoren sowie das Ansprechen nach Systemtherapie differenziert aufgreifen.

*Michael Oertel und Hans Theodor Eich, Münster*
